# Supplementary material for: A single-cell and spatial atlas of early human olfactory development
Source: Nat Commun. 2026 Apr 17;17:3537. doi: 10.1038/s41467-026-71595-6 (PMC13090377; doi:10.1038/s41467-026-71595-6)
Supplement: Supplementary file 1 — Supplementary information [file 41467_2026_71595_MOESM1_ESM.pdf]

Supplementary Information:

A Single-Cell and Spatial Atlas of Early Human Olfactory Development

Yvon Mbouamboua [1, 2*], Kevin Lebrigand [2*], Sreekala Nampoothiri [1], Marie Couralet [2], Marie-Jeanne Arguel [2], Ludovica Cotellessa [1], Cécile Allet [1], Vincent Prevot [1], Pascal Barbry [2*] and Paolo Giacobini [1*]

Corresponding authors: paolo.giacobini@inserm.fr; barbry@ipmc.cnrs.fr

Containing:

Supplementary figures 1 to 11

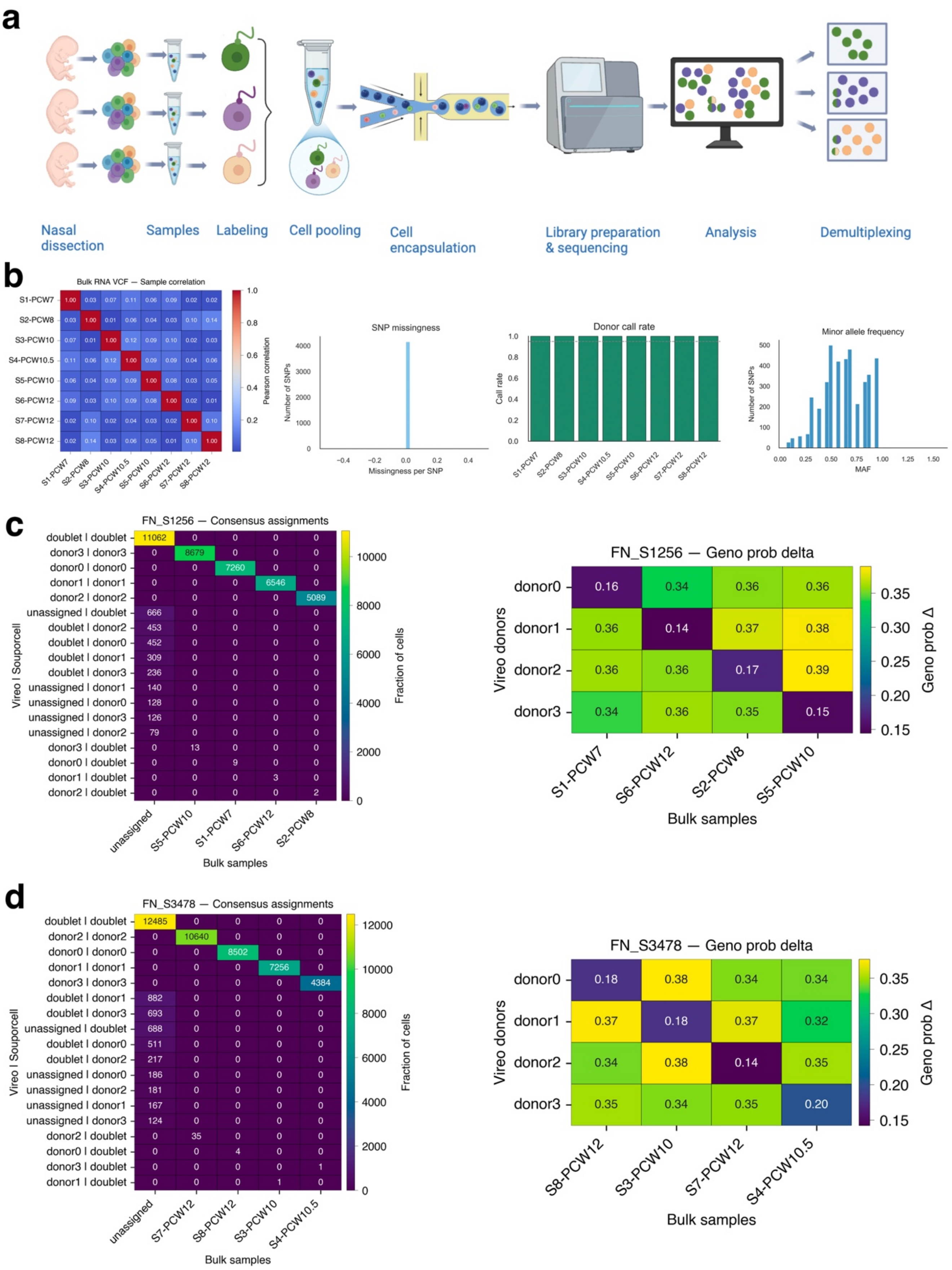
a
Nasal dissection
Samples
Labeling
Cell pooling
Cell encapsulation
Library preparation & sequencing
Analysis
Demultiplexing
b
Bulk RNA VCF — Sample correlation
S1-PCW7
S2-PCW8
S3-PCW10
S4-PCW10.5
S5-PCW10
S6-PCW12
S7-PCW12
S8-PCW12
Pearson correlation
SNP missingness
Number of SNPs
Missingness per SNP
Donor call rate
Call rate
Minor allele frequency
Number of SNPs
MAF
c
FN_S1256 — Consensus assignments
Vireo | Souporcell
Fraction of cells
Bulk samples
FN_S1256 — Geno prob delta
Vireo donors
Geno prob Δ
Bulk samples
d
FN_S3478 — Consensus assignments
Vireo | Souporcell
Fraction of cells
Bulk samples
FN_S3478 — Geno prob delta
Vireo donors
Geno prob Δ
Bulk samples

Supplementary Figure 1. Comparison of demultiplexing methods and individual assignments. (**a**) Schematic overview of the demultiplexing workflow, including nasal dissection, sample collection, labeling, cell pooling, cell encapsulation, library preparation, sequencing, analysis, and demultiplexing. Figure created in BioRender, and is licensed under CC BY 4.0 (https://BioRender.com/qpevax7). (**b**) Left to right: Pairwise sample correlation heatmap, Pearson correlation of genotypes across specimens. High values indicate consistent genotype calls between donors; lower values reflect specimen-specific differences or missing data. SNP missingness distribution, Fraction of missing genotypes per SNP in bulk RNA-seq VCF. SNPs with >20% missing were filtered prior to downstream analysis. Donor call rate, Fraction of genotypes successfully called per donor. Horizontal dashed line indicates expected quality threshold (95% call rate). Minor allele frequency (MAF) distribution, Distribution of SNPs passing filtering criteria (presence ≥ 80%, MAF ≥ 5%). (**c**) Donor-bulk genotype concordance across sequencing runs FN_S1256 and FN_S3478. Left: consensus heatmaps comparing single-cell donor assignments (Souporcell/Vireo) with bulk RNA-seq genotypes. Right: genotype probability delta (Δ) heatmaps computed using vireoSNP, illustrating donor separation. (**d**) Demultiplexing results for FN_S3478 shown as in (**c**). Rows correspond to combined donor labels and columns to bulk specimens; values indicate the number of cells assigned to each donor-bulk pairing. After excluding doublets and unassigned droplets, singlet assignments showed perfect concordance with bulk genotypes (accuracy = 100%, ARI = 1.0, Cohen's κ = 1.0), with no cross-donor misassignment across runs. Source data are provided with this paper.

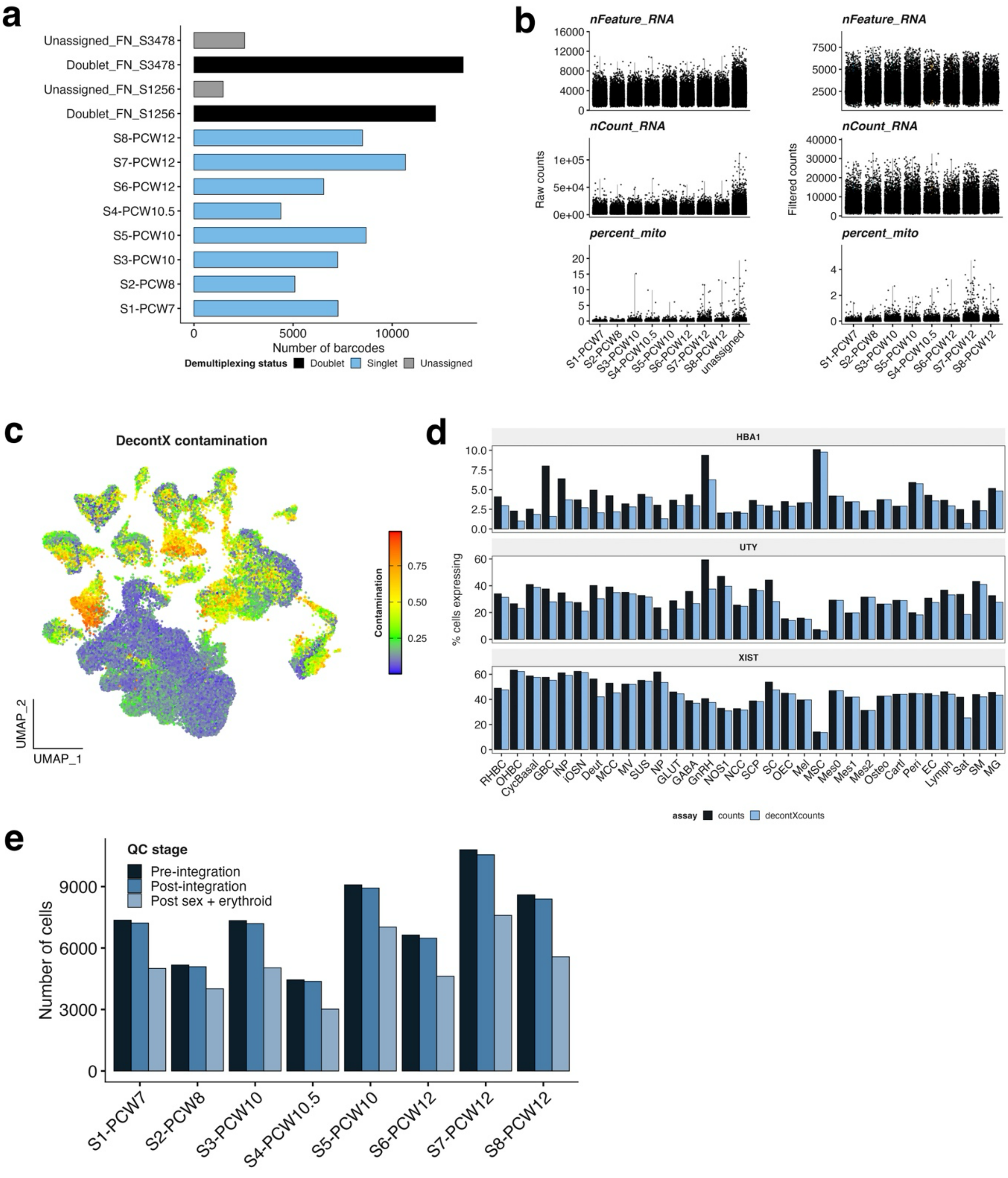

Supplementary Figure 2. Quality control and cell type annotation of human olfactory epithelium snRNA-seq data. (**a**), Demultiplexing results showing the number of singlets, doublets, and unassigned barcodes identified across specimens. (**b**), Distribution of quality control metrics before (left) and after (right) filtering, including the number of detected genes (nFeature_RNA), total UMI counts (nCount_RNA),

and mitochondrial read percentage (percent_mito). (**c**), UMAP embedding displaying DecontX-derived contamination scores, with cells colored by inferred contamination level. (**d**), expression of lineage and sex-associated markers (*HBA1*, *UTY*, *XIST*) across datasets, shown before and after DecontX correction. (**e**), Number of cells retained at successive quality control stages, pre-integration, post-integration, and post–sex and erythroid filtering for each specimen. Source data are provided as a Source Data File.

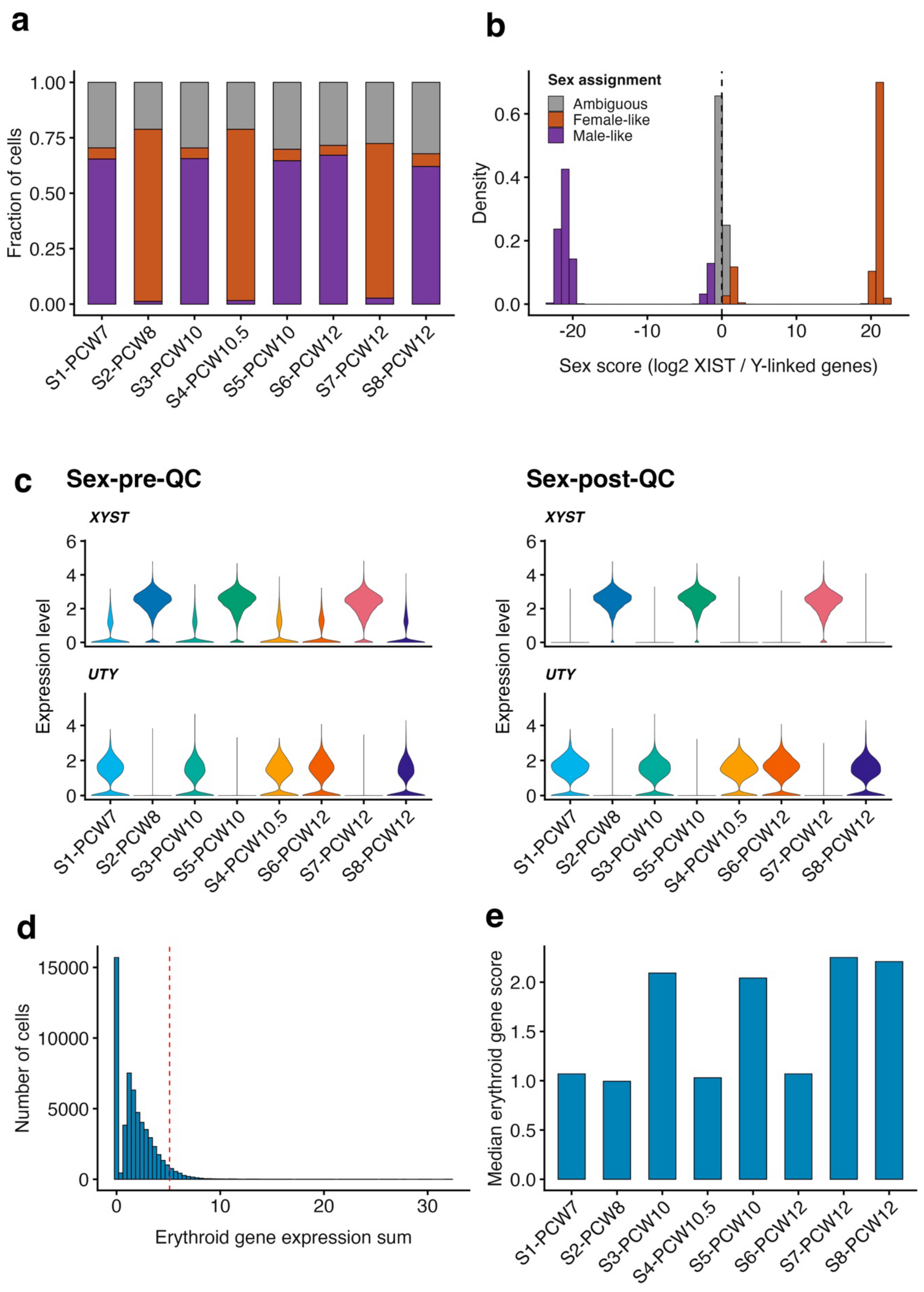

Supplementary Figure 3. Sex assignment quality control Detection of maternal erythroid contamination. (**a**) Stacked bar plot showing the fraction of cells classified as female-like, male-like, or ambiguous in each specimen. Sex assignment was based on per-cell sex scores derived from the $\log_2$

ratio of *XIST* expression to Y-linked gene expression. Fractions were computed after quality control and demultiplexing. (**b**) Distribution of per-cell sex scores computed as the $\log_2$ ratio of *XIST* expression to Y-linked gene expressions (*UTY*). Cells were classified as male-like, female-like, or ambiguous based on threshold score values. The dashed vertical line indicates a sex score of zero. (**c**) Violin plots of *XIST* and *UTY* expression across specimens before (left) and after (right) QC demonstrate removal of cells with discordant sex signatures, consistent with effective mitigation of ambient RNA and maternal blood contamination. (**d**) Distribution of erythroid gene expression scores across all cells; dashed line indicates the percentile-based threshold used to define erythroid-high cells. (**e**) Median erythroid score per specimen, indicating absence of specimen-specific bias. Cells with discordant sex assignment and elevated erythroid signal were removed prior to downstream analyses. Source data are provided as a Source Data File.

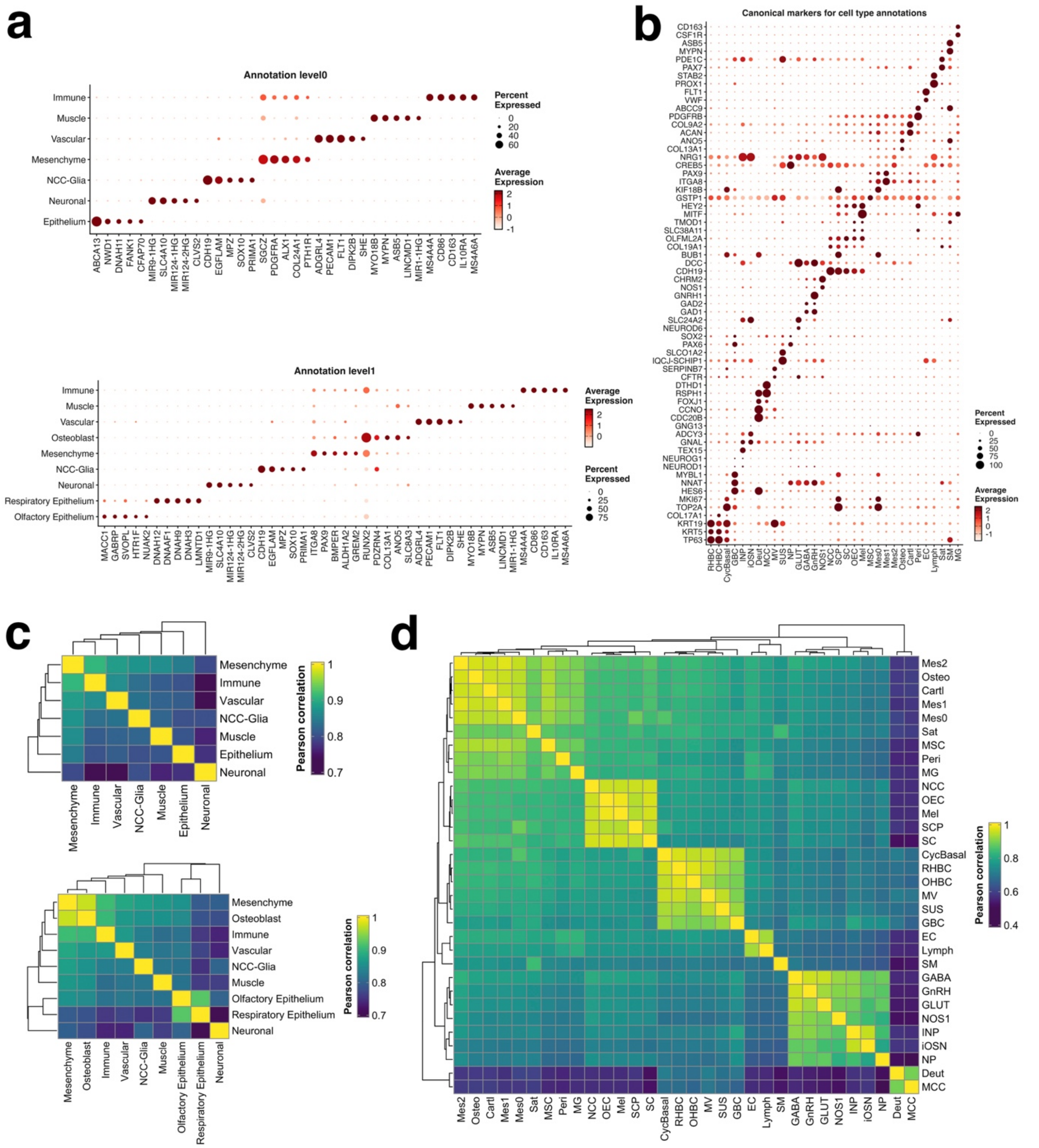

Supplementary Figure 4. Hierarchical annotation and transcriptional similarity of olfactory system cell types. (**a**) Dot plots showing top differentially expressed marker genes across two annotation levels: ann0 (broad cell classes) and ann1 (intermediate subtypes). Marker genes were identified using two-sided MAST tests on log-normalized counts, with Benjamini-Hochberg correction for multiple testing (padj < 0.05). Only genes with log-fold change > 0.25 and expressed in ≥ 25% of cells in at least one cluster were included. All dot plots were generated using this same MAST method. Dot size indicates the fraction of

cells expressing the gene, and color intensity reflects scaled expression per cell type. (**b**) Canonical markers used for manual cell type annotation. For each cluster, top markers ranked by average log_2 fold change are shown; dot size indicates the fraction of expressing cells, and color represents scaled expression. (**c, d**) Correlation heatmaps showing pairwise Pearson correlations of average gene expression between clusters at each annotation level, highlighting increased transcriptional specificity with hierarchical refinement. Data were derived from eight independent donors (PCW7-12).

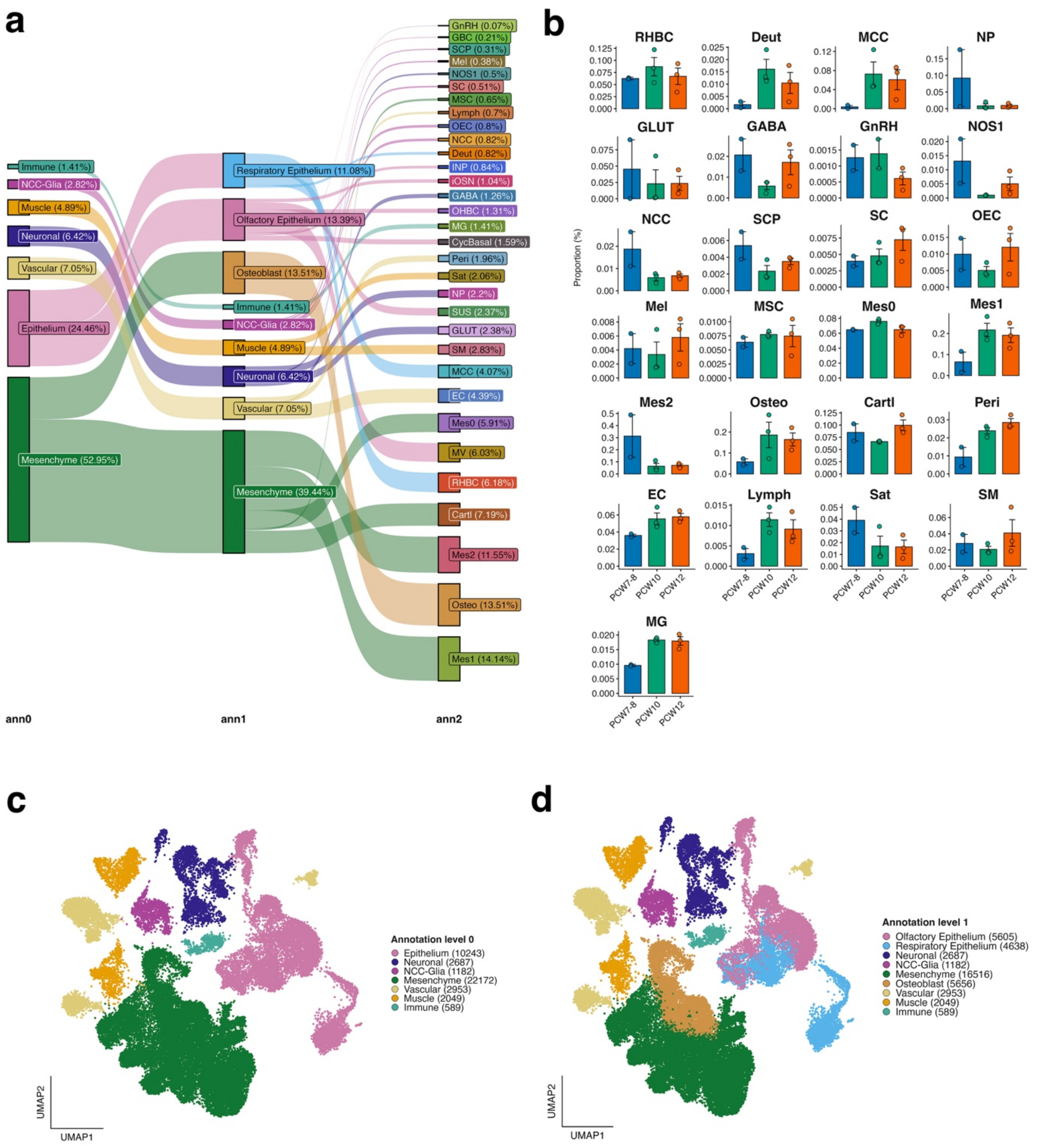

Supplementary Figure 5. Hierarchical annotation and compositional structure of olfactory epithelial cell populations. (**a**) Sankey diagram illustrates hierarchical relationships between annotation levels (ann0-ann2), with flow widths proportional to cell numbers and percentages indicated for each transition. (**b**) Bar plots of pseudobulk cell-type proportions per donor (n = 8) across developmental stages (post-conceptional weeks, PCW), after exclusion of olfactory epithelium cells. Bars represent the mean proportion per stage, error bars indicate SEM, and points show individual specimens (point outlines denote

specimen identity). Plots are facetted by cell type. Colors indicate developmental stage. (**c, d**) UMAP embeddings display global cellular organization colored by ann0 and ann1 annotations, revealing progressive refinement from broad cell classes to intermediate subtypes. Colors are consistent across panels consistent across panels. Source data are provided as a Source Data File.

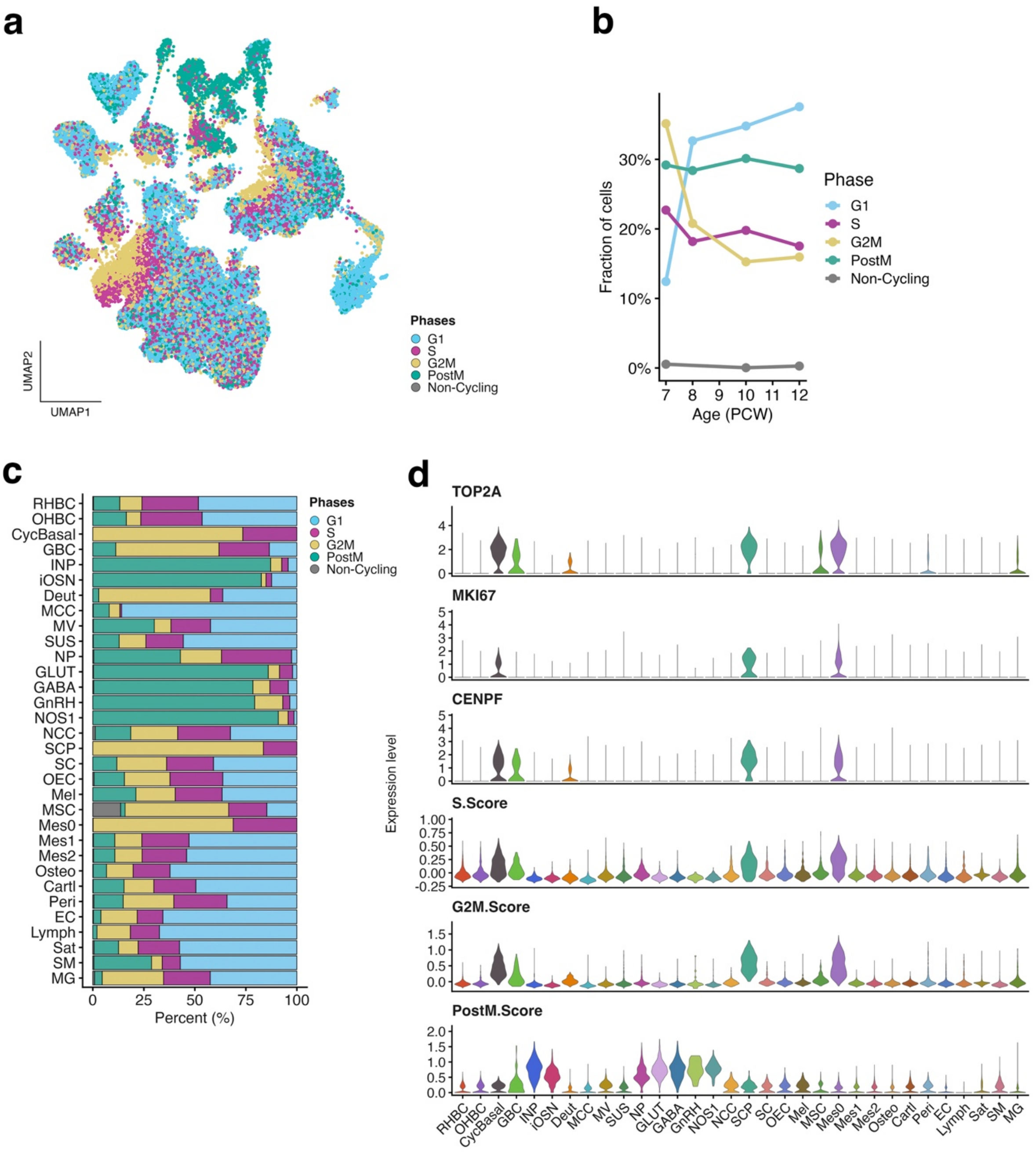

Supplementary Figure 6. Cell cycle dynamics in human olfactory epithelium cells. (**a**) UMAP visualization of single-nucleus transcriptomes colored by inferred cell cycle phase (G1, S, G2/M, post-mitotic). (**b**) Line plots depict temporal changes in phase distribution across PCW, emphasizing shifts from proliferative progenitors to post-mitotic differentiated cells. (**c**) Stacked bar plots show the proportion of cell types in each fetal stage (PCW7-12, *n* = 8). Data are presented as mean values +/- SEM across specimens (*n* = 8). Individual specimens are overlaid as points to show specimen-specific contributions. Fractions were calculated per specimen and log-transformed for visualization. (**d**) Violin plots display canonical cell cycle markers (*TOP2A, MKI67, CENPF*) alongside computed S, G2M, and post-mitotic

scores, capturing heterogeneity in cell cycle activity within distinct populations. Source data are provided as a Source Data File.

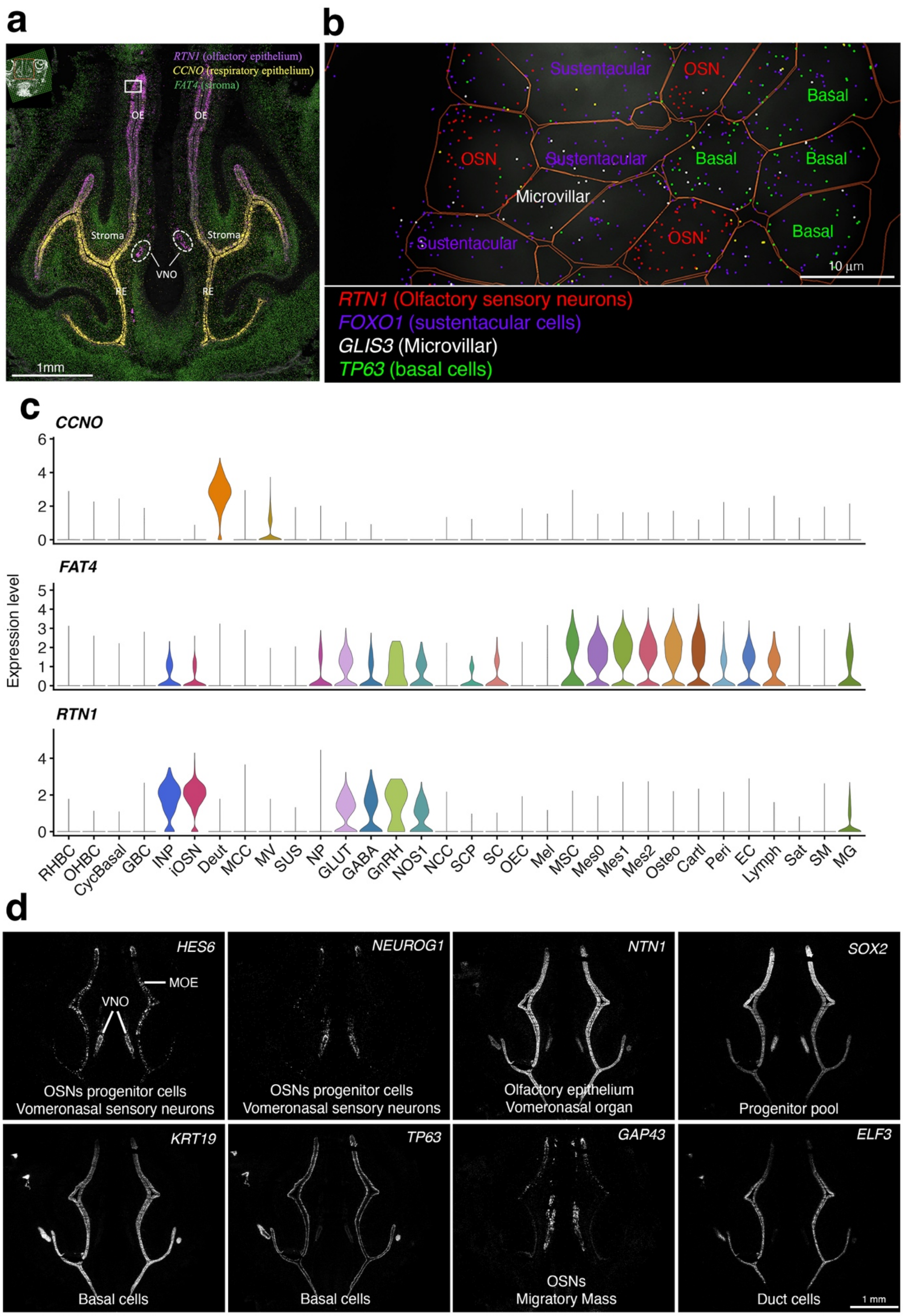
a
RTN1 (olfactory epithelium)
CCNO (respiratory epithelium)
FAT4 (stroma)
OE
OE
Stroma
Stroma
VNO
RE
RE
1mm
b
Sustentacular
OSN
Basal
OSN
Sustentacular
Basal
Basal
Microvillar
Sustentacular
OSN
Basal
10 µm
RTN1 (Olfactory sensory neurons)
FOXO1 (sustentacular cells)
GLIS3 (Microvillar)
TP63 (basal cells)
c
CCNO
FAT4
RTN1
Expression level
RHBC OHBC CycBasal GBC INP iOSN Deut MCC MV SUS NP GLUT GABA GnRH NOS1 NCC SCP SC OEC Mel MSC Mes0 Mes1 Mes2 Osteo Cartl Peri EC Lymph Sat SM MG
d
HES6
MOE
VNO
OSNs progenitor cells
Vomeronasal sensory neurons
NEUROG1
OSNs progenitor cells
Vomeronasal sensory neurons
NTN1
Olfactory epithelium
Vomeronasal organ
SOX2
Progenitor pool
KRT19
Basal cells
TP63
Basal cells
GAP43
OSNs
Migratory Mass
ELF3
Duct cells
1 mm

Supplementary Figure 7. Transcriptomic and spatial profiling of the developing olfactory epithelium. (**a**) MERFISH image of a PCW9 coronal nasal section showing *RTN1*, *CCNO*, and *FAT4* expression. (**b**) Higher-magnification view of the boxed region in (**a**) with cell segmentation by the Vizgen-Cellpose pipeline. *RTN1*, *FOXO1*, *GLIS3*, and *TP63* identify olfactory sensory neurons (OSNs), sustentacular, MV, and basal cells, respectively. (**c**) Violin plots of *CCNO*, *FAT4*, and *RTN1* expression across cell types. *CCNO* is enriched in Deuterosomal cells (Deut), *FAT4* shows broad expression, and *RTN1* marks neuronal lineages. (**d**) MERFISH validated the expression of marker genes defining OE cell populations, including progenitors (*SOX2*), OSN progenitors and vomeronasal neurons (*HES6*, *NEUROG1*), basal cells (*KRT19*, *TP63*), iOSN and migratory neurons (*GAP43*), and duct cells (*ELF3*), providing spatial validation of transcriptomic identities.

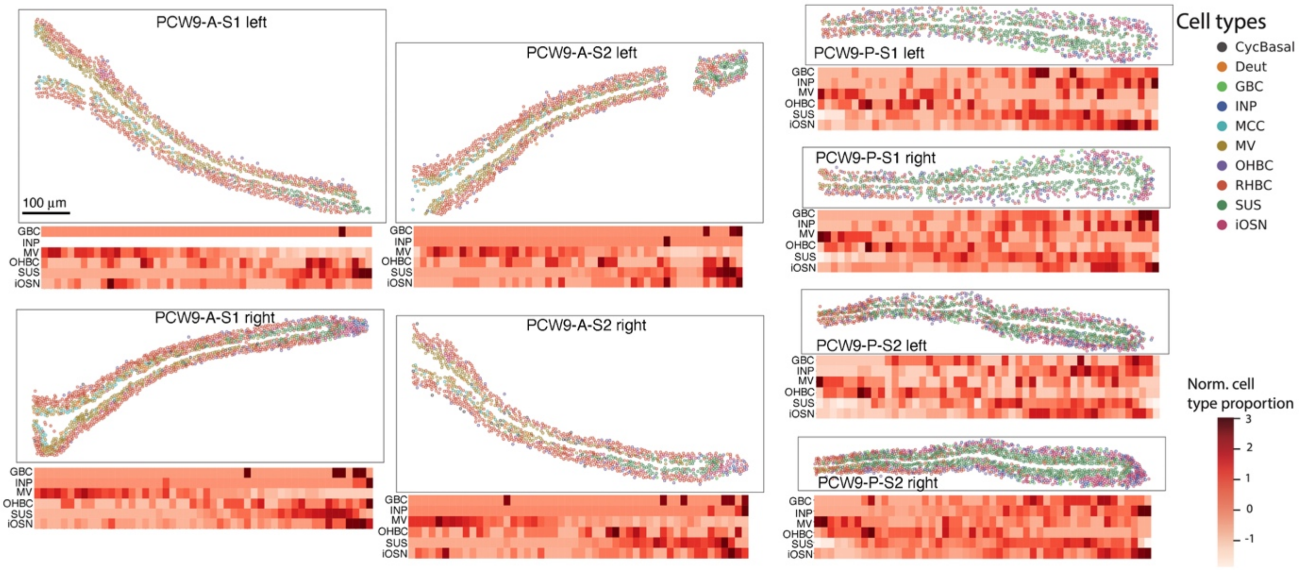

Supplementary Figure 8. Spatial distribution of major olfactory epithelium cell types across first-trimester specimens. Spatial transcriptomic maps showing annotated cell types along the left and right olfactory epithelial lamellae for PCW9-A-S1 (anterior section 1), PCW9-A-S2 (anterior section 2), PCW9-P-S1 (posterior section 1), and PCW9-P-S2 (posterior section 2) specimens. Each point represents a segmented cell colored by its predicted cell type of the OE, including globose basal cells (GBC), immediate neuronal precursors (INP), microvillar cells (MV), olfactory horizontal basal cells (OHBC), supporting cells (SUS), and immature olfactory sensory neurons (iOSN). Below each spatial map, heatmaps display the normalized proportion of each annotated cell type across spatial bins (50 µm bins), revealing conserved epithelial layering and specimen-specific variation in cell-type composition.

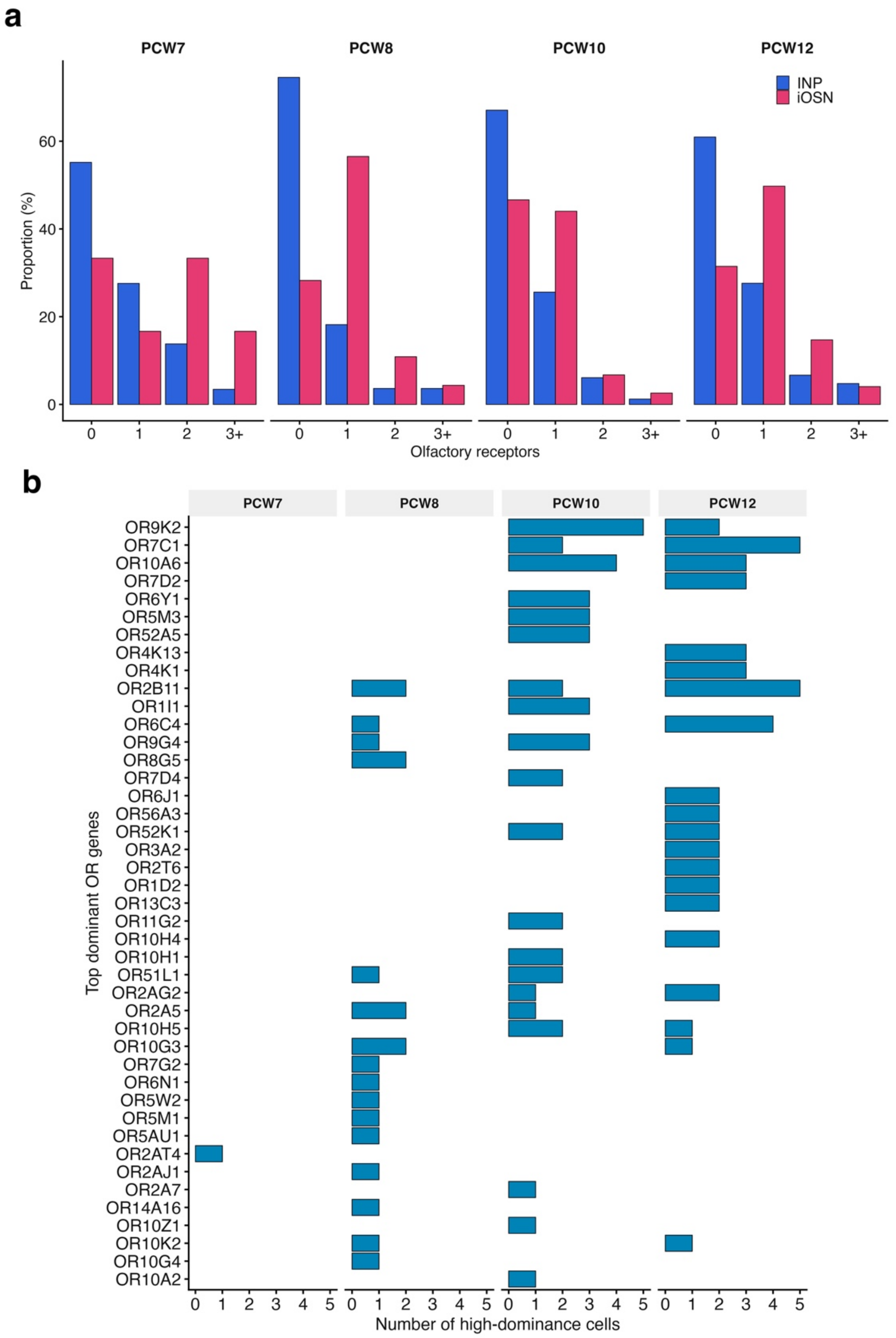

Supplementary Figure 9. Developmental activation of olfactory receptor programs in neuronal lineages. (**a**) Olfactory receptor (OR) gene-set activity in INP (immediate neuronal precursors) and iOSN (immature olfactory sensory neuron) across developmental stages (PCW7-12, *n* = 8). (**b**) Stage-specific dominant OR genes reveal progressive and lineage-restricted establishment of receptor expression, illustrating the maturation trajectory of human olfactory neurons. Source data are provided as a Source Data File.

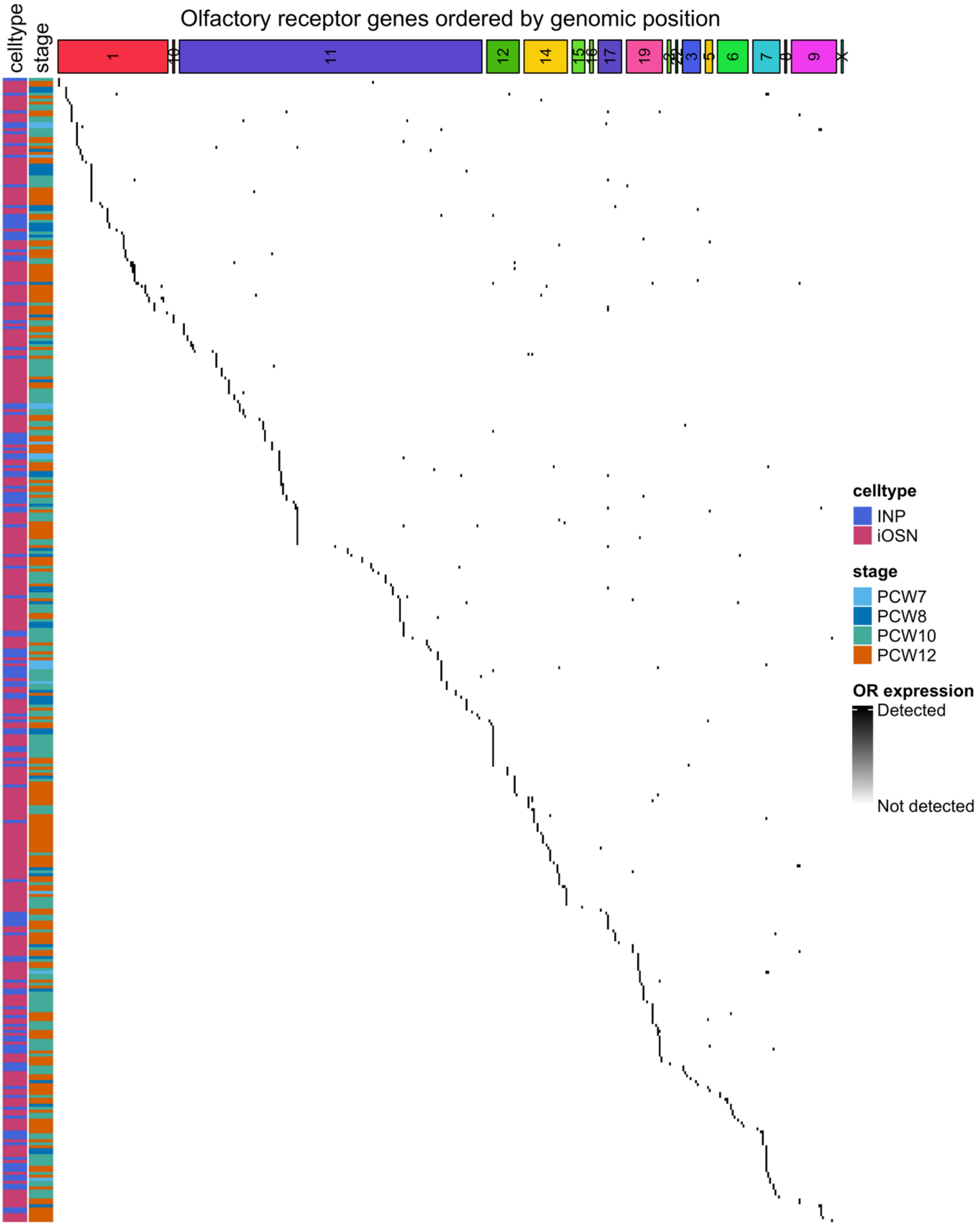

Supplementary Figure 10. Analysis of the expression of the olfactory receptors (ORs) in individual INPs and OSNs. Binary matrix showing olfactory receptor (OR) gene expression across individual cells. Rows represent single cells from different developmental stages (PCW7-12), color-coded by cell identity and age (left). Columns represent OR genes arranged by genomic position across chromosomes (top). Black pixels indicate detected OR expression. The diagonal pattern reflects mutually exclusive, dominant expression of a single OR per neuron, with sparse secondary OR expression.

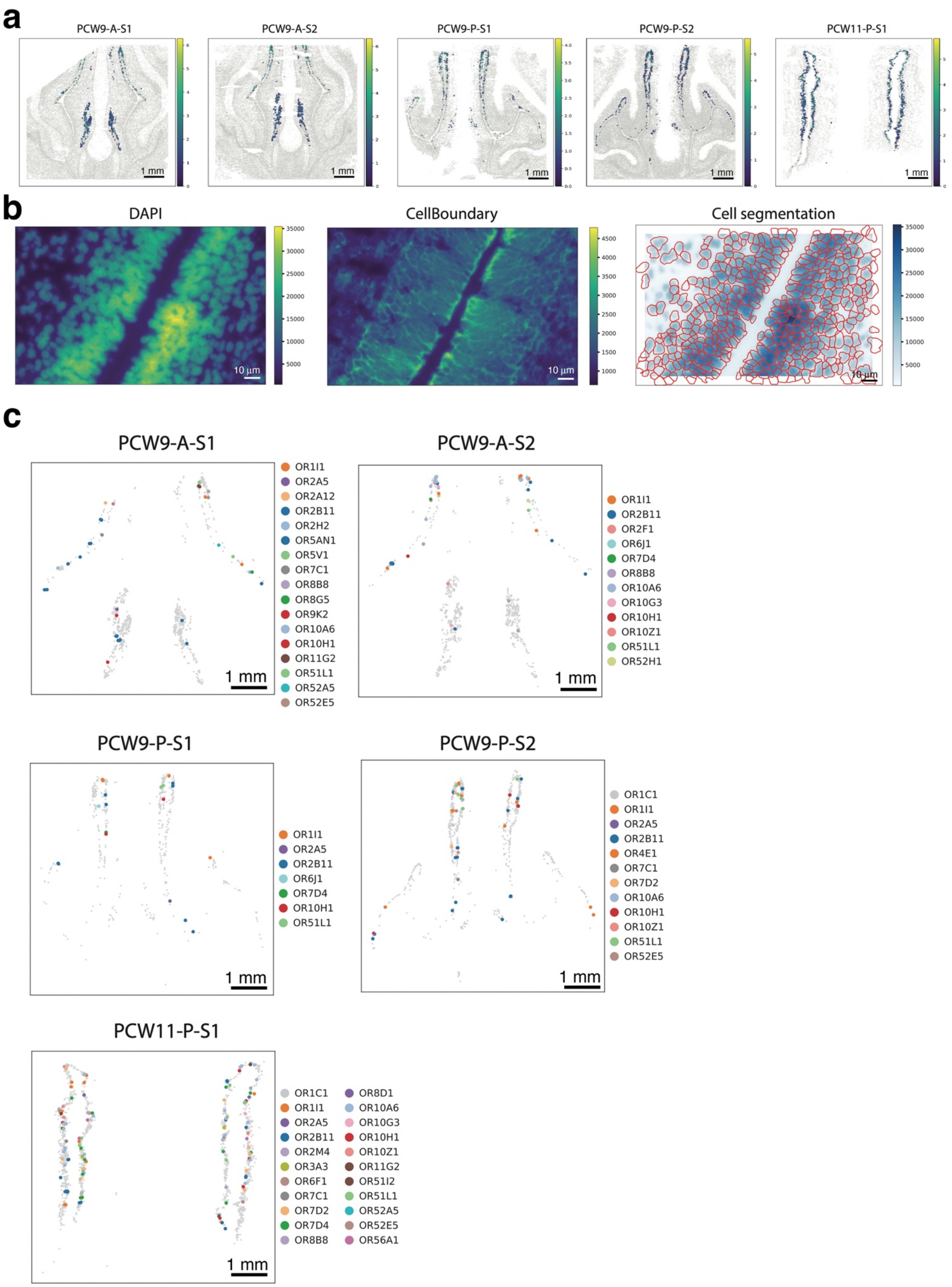
a
PCW9-A-S1
PCW9-A-S2
PCW9-P-S1
PCW9-P-S2
PCW11-P-S1
1 mm
b
DAPI
CellBoundary
Cell segmentation
10 μm
c
PCW9-A-S1
OR1I1
OR2A5
OR2A12
OR2B11
OR2H2
OR5AN1
OR5V1
OR7C1
OR8B8
OR8G5
OR9K2
OR10A6
OR10H1
OR11G2
OR51L1
OR52A5
OR52E5
PCW9-A-S2
OR1I1
OR2B11
OR2F1
OR6J1
OR7D4
OR8B8
OR10A6
OR10G3
OR10H1
OR10Z1
OR51L1
OR52H1
PCW9-P-S1
OR1I1
OR2A5
OR2B11
OR6J1
OR7D4
OR10H1
OR51L1
PCW9-P-S2
OR1C1
OR1I1
OR2A5
OR2B11
OR4E1
OR7C1
OR7D2
OR10A6
OR10H1
OR10Z1
OR51L1
OR52E5
PCW11-P-S1
OR1C1
OR1I1
OR2A5
OR2B11
OR2M4
OR3A3
OR6F1
OR7C1
OR7D2
OR7D4
OR8B8
OR8D1
OR10A6
OR10G3
OR10H1
OR10Z1
OR11G2
OR51I2
OR51L1
OR52A5
OR52E5
OR56A1
1 mm

Supplementary Figure 11. Spatial transcriptomic mapping of olfactory receptor (OR) gene expression in first-trimester human olfactory epithelium. (**a**) Spatial feature plots showing the aggregated expression of 57 OR genes across five specimens (PCW9-A-S1, PCW9-A-S2, PCW9-P-S1, PCW9-P-S2, and PCW11-P-S1). OR-expressing cells are concentrated along the presumptive olfactory epithelium, revealing conserved spatial organization across developmental stages and specimen replicates. (**b**) High-resolution image processing for cell segmentation. Left: DAPI staining used to identify nuclei. Middle: Cell boundary generated from the DAPI channel. Right: Resulting cell segmentation overlaid on the tissue section, highlighting individual cell outlines. (**c**) Visualization of top expressors iOSNs colored by their highly expressed OR.
