# Supplementary material for: A single-cell and spatial atlas of early human olfactory development
Source: Nat Commun. 2026 Apr 17;17:3537. doi: 10.1038/s41467-026-71595-6 (PMC13090377; doi:10.1038/s41467-026-71595-6)
Supplement: Supplementary file 2 — Description of Additional Supplementary Files [file 41467_2026_71595_MOESM2_ESM.pdf]

Description of Additional Supplementary Files

Supplementary Data 1. Demultiplexing and donor assignment concordance with bulk RNA-seq genotypes.

Single-cell donor assignments generated using Souporcell and Vireo are shown alongside consensus singlet classifications and corresponding bulk RNA-seq donor identities across two independent sequencing runs (FN_S1256 and FN_S3478). Droplets were classified as singlet, doublet, or unassigned based on combined demultiplexing and doublet detection results. After excluding doublets and unassigned droplets, high-confidence singlet assignments were compared to bulk genotypes. Donor labels showed perfect concordance with bulk RNA-seq identities (overall accuracy = 100%; Adjusted Rand Index = 1.0; Cohen's κ = 1.0), with no cross-donor misassignment across runs. Per-run concordance was 100% (FN_S1256: 27,596 cells; FN_S3478: 30,820 cells). The table includes cell barcodes, donor assignments from Souporcell and Vireo, droplet classifications, doublet scores (scds), consensus singlet assignments, matched bulk sample identity, and sequencing run information.

Supplementary Data 2. Per-donor cell contributions across cell clusters, sex, developmental stage, and individual samples.

The table summarizes the number (count) and percentage (pct) of cells assigned to each annotated cluster across the full dataset. For each cluster, counts are stratified by sex (male, female), developmental stage (PCW7–8, PCW10, PCW12), and individual samples (S1–S8). The final row indicates total cell numbers per stratification category (total cells = 41,875). This table provides a comprehensive overview of samples' representation and cellular composition across biological and developmental variables.

Supplementary Data 3. Sample-level sex assignment and erythroid contamination quality control in the developing human olfactory epithelium.

Per-nucleus sex was inferred using XIST-to–Y-linked gene expression ratios and classified as female-like, male-like, or ambiguous. The table summarizes, for each sample, total nuclei (n_cells), fractions of female-like, male-like, and ambiguous nuclei, median sex score, median erythroid gene expression score (eryth_sum), number and fraction of nuclei removed during quality control (n_removed, frac_removed), and the erythroid contamination threshold applied. Samples showed clear bimodal sex-score distributions consistent with sample's sex, with low erythroid scores supporting minimal maternal blood–derived ambient RNA contamination.

Supplementary Data 4. Differential expression of marker genes for cell annotation level 0.

Marker genes distinguishing clusters at annotation level 0 were identified using two-sided MAST tests, with multiple testing corrected using the Benjamini-Hochberg procedure (adjusted $P < 0.05$). Only genes with log-fold change > 0.25 and detected in ≥ 25% of cells in at least one cluster were included. The table provides gene identifiers, associated clusters, log-fold changes, adjusted *P* values, and detection fractions.

Supplementary Data 5. Differential expression of marker genes for cell annotation level 1.

Marker genes distinguishing clusters at annotation level 1 were identified using the same criteria as Table 4. Two-sided MAST tests with Benjamini-Hochberg correction (padj < 0.05), log-fold change > 0.25, and ≥ 25% detection in at least one cluster were applied. Columns include gene names, associated clusters, log-fold changes, adjusted *P* values, and detection fractions.

Supplementary Data 6. Differential expression of marker genes for cell annotation level 2.

Marker genes distinguishing clusters at annotation level 2 were identified with identical criteria to Tables 4 and 5. The table includes gene identifiers, cluster assignments, log-fold changes, adjusted *P* values, and detection fractions.

Supplementary Data 7. Inferred transcription factor regulon activity and lineage-specific regulatory programs across developing human olfactory epithelium cell states (PCW7-12).

Transcription factor (TF) regulon activity was inferred per cell cluster using single-cell regulatory network analysis to identify lineage-specific regulatory programs across developmental stages. The table lists, for each cluster and developmental stage (group), TFs, and their corresponding mean regulon activity scores. Positive scores indicate higher predicted activity, while negative scores indicate lower activity. These data support the identification of distinct regulatory programs underlying cell fate specification in the developing human olfactory epithelium.

Supplementary Data 8. All unique olfactory receptor (OR) genes detected in the fetal human olfactory epithelium and their per-cell expression.

This table lists all unique OR genes identified across the dataset, along with the number of cells expressing each gene (Nb cells expressing OR). Genes are ranked alphabetically, and the cell counts indicate the breadth of expression across olfactory epithelium cells. This dataset provides a comprehensive reference for OR gene detection frequency in the developing human olfactory system.

Supplementary Data 9. Classification of detected olfactory receptor (OR) genes by class and family in the developing human olfactory epithelium.

This table summarizes the distribution of OR genes detected in our dataset according to OR class (Class I vs. Class II) and OR family. For each family, the table lists the detected OR genes alongside the total number of OR genes in the human genome and the number of ORs detected in this study. This allows comparison of observed versus genomic repertoire and highlights the representation of different OR families across the developing human olfactory epithelium.

Supplementary Data 10. Per-cell olfactory receptor (OR) dominance scores and dominance bin assignments in olfactory epithelium cells.

This table provides cell-level information on OR expression dominance across olfactory epithelium (OE) cells. For each cell, the table includes the top-expressed OR gene and its expression, the second-highest OR expression, the calculated dominance score, and an adjusted score accounting for sparse expression. The number of expressed OR genes, cell type annotation (ann2), sample, developmental

stage, sex, and OE cell identity are provided. Cells are classified into dominance bins (≤ 0.5, 0.5-1, 1-1.5, 1.5-2, > 2) based on relative OR expression. A quality control flag indicates whether a cell had detectable top-expressed ORs. These data allow analysis of OR expression hierarchy and cell-level dominance patterns across the developing human olfactory epithelium.

Supplementary Data 11. Comprehensive list of genes and functional annotations identified in this study.

This table provides a complete catalog of genes analyzed in the developing human olfactory epithelium, including gene symbols, Ensembl identifiers (ensembl_id), full gene names, and concise functional descriptions.
