# Supplementary material for: A single-cell and spatial atlas of early human olfactory development
Source: Nat Commun. 2026 Apr 17;17:3537. doi: 10.1038/s41467-026-71595-6 (PMC13090377; doi:10.1038/s41467-026-71595-6)
Supplement: Supplementary file 4 — Reporting Summary [file 41467_2026_71595_MOESM4_ESM.pdf]

nature portfolio

Corresponding author(s): Paolo Giacobini and Pascal Barbry

Last updated by author(s): 24/02/2026

Reporting Summary

Nature Portfolio wishes to improve the reproducibility of the work that we publish. This form provides structure for consistency and transparency in reporting. For further information on Nature Portfolio policies, see our Editorial Policies and the Editorial Policy Checklist.

Statistics

For all statistical analyses, confirm that the following items are present in the figure legend, table legend, main text, or Methods section.

n/a	Confirmed	
☐	☒	The exact sample size (*n*) for each experimental group/condition, given as a discrete number and unit of measurement
☐	☒	A statement on whether measurements were taken from distinct samples or whether the same sample was measured repeatedly
☐	☒	The statistical test(s) used AND whether they are one- or two-sided<br>*Only common tests should be described solely by name; describe more complex techniques in the Methods section.*
☒	☐	A description of all covariates tested
☐	☒	A description of any assumptions or corrections, such as tests of normality and adjustment for multiple comparisons
☐	☒	A full description of the statistical parameters including central tendency (e.g. means) or other basic estimates (e.g. regression coefficient) AND variation (e.g. standard deviation) or associated estimates of uncertainty (e.g. confidence intervals)
☐	☒	For null hypothesis testing, the test statistic (e.g. *F*, *t*, *r*) with confidence intervals, effect sizes, degrees of freedom and *P* value noted<br>*Give P values as exact values whenever suitable.*
☒	☐	For Bayesian analysis, information on the choice of priors and Markov chain Monte Carlo settings
☒	☐	For hierarchical and complex designs, identification of the appropriate level for tests and full reporting of outcomes
☐	☒	Estimates of effect sizes (e.g. Cohen's *d*, Pearson's *r*), indicating how they were calculated

Our web collection on statistics for biologists contains articles on many of the points above.

Software and code

Policy information about availability of computer code

Data collection	Data were collected on the following instruments: Chromium Next GEM Single Cell 3' v3.1, NextSeq 2000 Illumina platform, Vizgen MERSCOPE platform (Vizgen, BP0939 #10400002), Agilent 2100 Bioanalyzer, CM3050 Leica cryostat, laser scanning ZEISS LSM 710 AiryScan confocal microscope.
Data analysis	Software and tools used for data analysis: Photoshop (version 26.0.0, Adobe Systems, San Jose, USA), Cell Ranger software v6.0.0, STAR aligner, Seurat R package v4.0, R v4.0.2, Demuxafy, clusterProfiler R package, Slingshot (v2.6.0) , DecoupleR R package (v.2.12.0), Vizgen Post-processing Tool, scMusketteers algorithm, Vireo, Souporcell , Single-Cell Doublet Scorer, CellPose.

For manuscripts utilizing custom algorithms or software that are central to the research but not yet described in published literature, software must be made available to editors and reviewers. We strongly encourage code deposition in a community repository (e.g. GitHub). See the Nature Portfolio guidelines for submitting code & software for further information.

Data

Policy information about availability of data

All manuscripts must include a data availability statement. This statement should provide the following information, where applicable:

- Accession codes, unique identifiers, or web links for publicly available datasets
- A description of any restrictions on data availability
- For clinical datasets or third party data, please ensure that the statement adheres to our policy

All data generated or analyzed in the current study are included in the article and its Suppl. Figures and Suppl. Data. The raw snRNA-seq datasets generated during

the current study have been deposited in the European Genome/Phenome Archive (https://ega-archive.org/datasets/EGAD50000001712). Access to EGA archive datasets is obtained by formal application to the Data Access Committee (DAC). Each DAC requires users/applicants to sign a Data Access Agreement (DAA), which details the terms and conditions of use for each dataset. An interactive Shiny application associated with processed human snRNA-seq data has been deposited on Zenodo (https://doi.org/10.5281/zenodo.18245692). MERFISH data have been deposited in Gene Expression Omnibus under accession code GSE303809 [https://www.ncbi.nlm.nih.gov/geo/query/acc.cgi?acc=GSE303809]. Source data are provided with this paper.

Custom scripts and codes used for snRNA-seq analysis can be found at https://github.com/ymbouamboua/HuDeCa. Python scripts for re-analysis and figures production of MERFISH experiments can be found at https://github.com/cobioda/human_fetal_olfactory_system.

Research involving human participants, their data, or biological material

Policy information about studies with human participants or human data. See also policy information about sex, gender (identity/presentation), and sexual orientation and race, ethnicity and racism.

Reporting on sex and gender	Potential sex differences were considered and analyzed in this work. Our analysis revealed no obvious sex-differences in the cell-types (Wilcoxon rank-sum test P > 0.05; Fig. 1e, Suppl Data Table 1).
Reporting on race, ethnicity, or other socially relevant groupings	NA
Population characteristics	NA
Recruitment	NA
Ethics oversight	Authorization to use human tissues was granted by the French agency for biomedical research (Agence de la Biomédecine, Saint-Denis La Plaine, France; N° PFS19-012) and the INSERM Ethics Committee (IRB00003888).

Note that full information on the approval of the study protocol must also be provided in the manuscript.

Field-specific reporting

Please select the one below that is the best fit for your research. If you are not sure, read the appropriate sections before making your selection.

[x] Life sciences [ ] Behavioural & social sciences [ ] Ecological, evolutionary & environmental sciences

For a reference copy of the document with all sections, see nature.com/documents/nr-reporting-summary-flat.pdf

Life sciences study design

All studies must disclose on these points even when the disclosure is negative.

Sample size	Sample sizes were based on previous studies using similar models and on the documentation of similar well-characterized experiments in the literature in vivo. No statistical methods were used to pre-determine sample sizes giving the nature and scarcity of this kind of samples (human fetal samples). Sample sizes are provided in the main text, methods section and figure legends.
Data exclusions	Allele concordance between single-nucleus genotypes and bulk variants was computed, and donor assignments were classified as mapped, ambiguous, or rejected based on posterior donor genotype probability separation (delta genotype probability), as implemented in Vireo, using thresholds of minimum concordance ≥0.60 and probability delta ≤0.05. Doublets and unassigned nuclei were removed, retaining high-confidence singlets for downstream analysis.
Replication	As it is typical in this field, single-nuclei RNA-sequencing and spatial transcriptomics data were not replicated due to limited tissue samples and cost of the experiments.
Randomization	Considering the exploratory nature of the study, randomization was not relevant.
Blinding	Investigators were not blinded to group allocations since this information was required to select the samples for the study and to design and guide analyses of snRNAseq and spatial transcriptomics.

Reporting for specific materials, systems and methods

We require information from authors about some types of materials, experimental systems and methods used in many studies. Here, indicate whether each material, system or method listed is relevant to your study. If you are not sure if a list item applies to your research, read the appropriate section before selecting a response.

Materials & experimental systems

n/a	Involved in the study	
☐	☒	Antibodies
☒	☐	Eukaryotic cell lines
☒	☐	Palaeontology and archaeology
☒	☐	Animals and other organisms
☒	☐	Clinical data
☒	☐	Dual use research of concern
☒	☐	Plants

Methods

n/a	Involved in the study	
☒	☐	ChIP-seq
☒	☐	Flow cytometry
☒	☐	MRI-based neuroimaging

Antibodies

Antibodies used

We used the following primary antibodies: anti-Olig2 goat IgG (R&D Systems, #AF2418, diluted at 1/100), anti-Tubulin beta III, TUJ1, mouse monoclonal (Biolegend, #801201, diluted at 1/200), anti-SOX2 (Y-7) goat polyclonal (Santa Cruz, # sc-17320, diluted at 1/200) and anti-Cytokeratin 5 (EP1601Y) rabbit monoclonal (Abcam, # ab52635, diluted at 1/500). The secondary antibodies used were the Alexa Fluor 568 Donkey anti-Goat IgG 1:400 (ThermoFisher, # A-11057), Alexa Fluor 488 Donkey anti-Goat IgG 1:400 (ThermoFisher, #, 11055), Alexa Fluor 647-conjugated Donkey anti-Mouse 1:400 (ThermoFisher, # A-31571) and Alexa Fluor 568 Donkey anti-Rabbit IgG 1:400 (ThermoFisher, # A-10042).

Validation

All antibodies utilized are commercially available and validated by the manufactures (https://www.rndsystems.com; https://www.biolegend.com; https://www.scbt.com; https://www.abcam.com; https://www.thermofisher.com)

Plants

Seed stocks

Report on the source of all seed stocks or other plant material used. If applicable, state the seed stock centre and catalogue number. If plant specimens were collected from the field, describe the collection location, date and sampling procedures.

Novel plant genotypes

Describe the methods by which all novel plant genotypes were produced. This includes those generated by transgenic approaches, gene editing, chemical/radiation-based mutagenesis and hybridization. For transgenic lines, describe the transformation method, the number of independent lines analyzed and the generation upon which experiments were performed. For gene-edited lines, describe the editor used, the endogenous sequence targeted for editing, the targeting guide RNA sequence (if applicable) and how the editor was applied.

Authentication

Describe any authentication procedures for each seed stock used or novel genotype generated. Describe any experiments used to assess the effect of a mutation and, where applicable, how potential secondary effects (e.g. second site T-DNA insertions, mosiacism, off-target gene editing) were examined.
